# Supplementary data of “Impacts of mesic and xeric urban vegetation on outdoor thermal comfort and microclimate in Phoenix, AZ”

**DOI:** 10.1016/j.dib.2015.11.006

**Published:** 2015-11-10

**Authors:** Jiyun Song, Zhi-Hua Wang

**Affiliations:** School of Sustainable Engineering and the Built Environment, Arizona State University, United States

## Abstract

An advanced Markov-Chain Monte Carlo approach called Subset Simulation is described in Au and Beck (2001) [1] was used to quantify parameter uncertainty and model sensitivity of the urban land-atmospheric framework, viz. the coupled urban canopy model-single column model (UCM-SCM). The results show that the atmospheric dynamics are sensitive to land surface conditions. The most sensitive parameters are dimensional parameters, i.e. roof width, aspect ratio, roughness length of heat and momentum, since these parameters control the magnitude of sensible heat flux. The relative insensitive parameters are hydrological parameters since the lawns or green roofs in urban areas are regularly irrigated so that the water availability for evaporation is never constrained.

**Specifications Table**

**Table:** 

Subject area	Climatology
More specific subject area	Urban microclimate
Type of data	Table, text file
How data was acquired	Numerical simulation by an advanced Markov-Chain Monte Carlo approach
Data format	Analyzed
Experimental factors	The meteorological data was obtained from Eddy Covariance Measurement Tower and was kept constant for different landscaping scenarios.
Experimental features	We changed different land surface parameters in different scenarios and compared the sensitivity of these surface parameters to the evolution of atmospheric variables (i.e. boundary layer height, temperature, humidity).
Data source location	Phoenix, Arizona, US (33.48 N, 112.14 W)
Data accessibility	Data is with this article.

## Value of the data

• An advanced stochastic approach has been used to simulate extremes of urban microclimate.
• The urban boundary layer (UBL) is sensitive to urban surface state as the lower boundary condition.
• Urban form and design is highly related to urban boundary layer dynamics.

## 1 Experimental design, materials and methods

To quantify the numerical sensitivity of the UCM-SCM framework, we used an advanced Markov-Chain Monte Carlo (MCMC) procedure, known as the Subset Simulations [1]. This approach breaks down statistically extreme events with small exceedance probability into a sequence of more frequent events by introducing intermediate exceedance events. The targeted small exceedance probability is then expressed as a product of larger conditional probabilities of each intermediate event. In addition, MCMC technique is adopted based on effective accept/reject rules in Subset Simulations to improve computational efficiency. For example, a rare event with target exceedance probability of *p*_*f*_=10^−4^ (i.e. the probability of occurrence is less than 1 in 10,000), can be effectively broken down into 4 different sampling, including the first level of unconditional direct Monte Carlo simulation following a prescribed probability distribution function (PDF) and 3 subsequent conditional MCMC levels, each sample with a moderate conditional probability of *p*_0_=0.1.

For better quantification, a percentage sensitivity index (PSI) devised in [2] is adopted here to measure the model sensitivity to an uncertain parameter *X* by calculating the average deviation of conditional sample means to that of the original PDF:

(1) $$\mathrm{PSI}[X]=\frac{1}{N}\sum_{i=1}^{N}\frac{E\left[X|Y>y_{i}\right]-E\left[X\right]}{E\left[X\right]},$$

where *i* is the conditional (MCMC) level index, *N*=3 the total conditional levels, *E*[*X*] the statistical mean (expected value) of the original unconditional distribution in level 0, *E*[*X|Y>y*_*i*_] the mean value of *X* at conditional level *i*, *Y* the value of monitored model response, and *y*_*i*_ the threshold values at exceedance probability of each intermediate level *i*. The magnitude of PSI quantifies the significance of sensitivity, while the sign of PSI indicates the correlation between monitored output *Y* and input parameter *X*, i.e. positive PSI means increasing *X* will lead to an increase of output *Y* and negative PSI means increasing *X* will lead to a decreased *Y*.

The error analysis of key input parameters for critical model outputs (UBL height *z*_*h*_, virtual potential temperature *θ*_*v*_, and specific humidity *q* in the mixed layer) under different surface greening scenarios with different vegetation fraction *f*_*veg*_ is presented in Table 1. Three groups of model inputs were tested, including (1) hydrological parameters (saturated soil water content *W*_*s*_, residual soil water content *W*_*r*_, and saturated hydraulic conductivity *K*_*s*_); (2) dimensional parameters (roof width *r*, building aspect ratio *h*/*w* with *w* the road width, and roughness lengths of momentum *Z*_*m,v*_ and *Z*_*m,b*_ above vegetated and built surfaces); (3) atmospheric parameters controlling the upper boundary conditions of the UBL (entrainment rate at the inversion *w*_*e*_, lapse rate in the free atmosphere *γ*_*θv*_). It can be shown that the model predictions of UBL dynamics are in general most sensitive to dimensional parameters, and relatively insensitive to hydrological parameters. This implicates that urban form and design can significantly alter UBL dynamics and the effect of regularly irrigated urban vegetation on the UBL is steady.

## Figures and Tables

**Table 1:** Estimates of PSI values for critical UBL height *z*_*h*_, virtual potential temperature *θ*_*v*_, and specific humidity *q* in the mixed layer.

Input parameters	*z*_*h*_			*θv*			*q*		
	*f*_*veg*_=0	0.5	0	*f*_*veg*_=0	0.5	1	*f*_*veg*_=0.1	0.5	1

Hydrological									
*W*_*s*_	0.35	−1.36	0.24	−0.04	0.34	−0.17	−0.49	1.39	1.84
*W*_*r*_	1.63	1.41	2.70	0.44	0.22	2.26	3.59	1.11	1.00
*K*_*s*_	−0.17	−2.48	0.64	0.24	−1.59	−1.89	−0.90	0.53	−1.94
Dimensional									
*r*	29.73	5.59	−25.08	34.60	2.65	−26.72	27.95	33.55	37.17
*h*/*w*	−33.23	−69.41	−87.32	−40.08	−79.89	−89.15	3.95	7.55	5.86
*Z*_*m,b*_	38.53	34.42	1.00	42.58	24.38	−1.13	−9.76	−7.75	7.72
*Z*_*m*_*,*_*v*_	0.26	−0.31	−5.36	0.53	−1.00	−0.26	−15.41	−16.77	−1.28
Atmospheric									
*w*_*e*_	20.16	19.78	10.91	−14.61	−14.93	−6.65	−1.45	−4.03	−1.55
*γ*_*θv*_	−33.46	−32.33	−19.91	27.22	22.62	12.84	6.20	5.20	6.12
